# Brain metastases in lung adenocarcinoma: impact of EGFR mutation status on incidence and survival

**DOI:** 10.2478/raon-2014-0016

**Published:** 2014-04-25

**Authors:** Karmen Stanic, Matjaz Zwitter, Nina Turnsek Hitij, Izidor Kern, Aleksander Sadikov, Tanja Cufer

**Affiliations:** 1 Department of Radiotherapy, Institute of Oncology Ljubljana, Ljubljana, Slovenia; 2 Faculty of Medicine, University of Maribor, Slovenia; 3 University Clinic Golnik, Slovenia; 4 Faculty of Computer and Information Science, University of Ljubljana, Slovenia

**Keywords:** brain metastases, lung adenocarcinoma, EGFR mutations

## Abstract

**Background:**

The brain represents a frequent progression site in lung adenocarcinoma. This study was designed to analyse the association between the epidermal growth factor receptor (EGFR) mutation status and the frequency of brain metastases (BM) and survival in routine clinical practice.

**Patients and methods:**

We retrospectively analysed the medical records of 629 patients with adenocarcinoma in Slovenia who were tested for EGFR mutations in order to analyse the cumulative incidence of BM, the time from the diagnosis to the development of BM (TDBM), the time from BM to death (TTD) and the median survival.

**Results:**

Out of 629 patients, 168 (27%) had BM, 90 patients already at the time of diagnosis. Additional 78 patients developed BM after a median interval of 14.3 months; 25.8 months in EGFR positive and 11.8 months in EGFR negative patients, respectively (p = 0.002). EGFR mutations were present in 47 (28%) patients with BM. The curves for cumulative incidence of BM in EGFR positive and negative patients demonstrate a trend for a higher incidence of BM in EGFR mutant patients at diagnosis (19% *vs.* 13%, p = 0.078), but no difference later during the course of the disease. The patients with BM at diagnosis had a statistically longer TTD (7.3 months) than patients who developed BM later (3.1 months). The TTD in EGFR positive patients with BM at diagnosis was longer than in EGFR negative patients (12.6 *vs.* 6.8, p = 0.005), while there was no impact of EGFR status on the TTD of patients who developed BM later.

**Conclusions:**

Except for a non-significant increase of frequency of BM at diagnosis in EGFR positive patients, EGFR status had no influence upon the cumulative incidence of BM. EGFR positive patients had a longer time to CNS progression. While EGFR positive patients with BM at diagnosis had a longer survival, EGFR status had no influence on TTD in patients who developed BM later during the course of disease.

## Introduction

The brain represents a frequent progression site in lung adenocarcinoma.^1^,^2^ The incidence of BM is increasing, probably due to a better treatment and prolonged survival as well as due to better imaging techniques.^3^ This condition is often disabling and reduces the patients’ quality of life.

The survival, even after aggressive multimodality treatment, remains poor.^4^ Therefore new subgroups that might benefit from new treatments are being identified.^5^,^6^ In the last few years, much effort and research in lung cancer has been oriented to molecular targets, *e.g*. EGFR activating mutations. Although a substantial proportion of patients with EGFR mutated tumours develop BM, the prevalence and the best treatment options for progression to the central nervous system (CNS) have not yet been finally determined.

Prospective trials for progression to CNS are challenging to conduct, therefore retrospective analyses and meta-analyses remain useful and important research tools.

The aim of our retrospective study was to determine the frequency of BM at diagnosis and during the course of disease, the time to the development of BM and the survival after the diagnosis of BM in relation to EGFR mutation status.

## Patients and methods

### Patients

Between December 2009 and January 2012, 804 patients with lung cancer in Slovenia were tested for EGFR mutations. After excluding cases with other histologic types, 629 patients with primary lung adenocarcinoma and with a definitive report on the mutation status were selected for this analysis.

The patients included in this analysis had a specific oncological treatment at the Institute of Oncology, the University Clinic Golnik and the University Clinic Maribor. The testing was performed either as a routine procedure of adenocarcinoma at the time of diagnosis or, upon a special request of the treating oncologist, in patients who were diagnosed in the past or were candidates for the treatment with TKI at relapse. The medical records of patients were reviewed retrospectively.

The TNM staging is based on 7^th^ Edition. All patients diagnosed before 2010 were restaged according to the new classification.^7^

The presence of BM was diagnosed with computed tomography (CT) or brain magnetic resonance imaging (MRI) either within the initial diagnostic staging of lung cancer or when patients became symptomatic.

The following parameters were recorded: demographic and clinical characteristics, the date of diagnosis, TNM classification, treatment characteristics, the date of first progression after primary treatment, the date of first BM; extracranial sites of disease activity at the time of BM diagnosis or any progression, the treatment of BM, the time of death or the last follow up. The smoking status was categorised as follows: nonsmokers (< 100 cigarettes in their lifetime), former smokers (stopped > 1 year before diagnosis of lung cancer), or current smokers. Performance status (PS) ranged from 0 to 4 according to Eastern Cooperative Oncology Group (ECOG) criteria. Weight loss of more than 2 kilograms per month before diagnosis of lung cancer was considered important. The follow up took place through 7th October 2013.

### EGFR testing

There was no extra testing performed only for the purpose of this retrospective analysis. Pathological expertize and results of molecular testing were used for analysis. The samples used to extract genomic DNA were either from formalin-fixed, paraffin-embedded tissue sections or cytological slide preparations. The quantification of extracted DNA was done on Qubit Fluorometer (Invitrogen, Carlsbad, USA). To detect EGFR gene activating mutations, the samples were tested with TheraScreen EGFR29 Mutation Kit (DxS Diagnostics, Qiagen, Manchester, UK).

### Statistical analysis

The primary endpoints in this analysis were the cumulative incidence of BM, the time to the development of brain metastases (TDBM) and the survival after the diagnosis of BM representing the time to death (TTD). The TDBM was calculated from the time of the diagnosis to the time of the development of BM for all patients who had no BM at diagnosis. The TTD was calculated from the date of BM to the date of death from any cause or the date of the last follow-up; censored observations represent patients alive at the time of the last follow-up. The secondary endpoint of this analysis was the overall survival (OS) calculated from the date of diagnosis to the date of death due to any cause. The Kaplan-Meier (KM) method and the log rank test were used to test for the difference between EGFR positive and negative patients. The cumulative incidence was calculated using 1-KM, using progression to CNS as an event. The association between the EGFR mutation status and the clinico-pathological characteristics of patients were tested using the Mann-Whitney U (MW-U) or the Kruskal Wallis H (KW-H) test. All p values reported were based on the two-sided hypothesis. The statistical analysis was computed using SPSS v.20 statistical package.

This survey was approved by the National Ethics Committee on 18.10.2011, No.143/1.

## Results

### Patient characteristics

The baseline characteristics of all 629 adenocarcinoma patients are presented in Table 1. The series included 326 (52%) men and 303 (48%) women with a median age of 64 years (range from 25 to 88). All patients were Caucasians. A statistically significant higher proportion of EGFR positive patients was among women (67.9% *vs.* 32.1%), p < 0.001 and nonsmokers (60.6% *vs.* 33.6%), p < 0.001. Out of 629 patients included in the analysis, 379 (60%) had a metastatic disease. Ninety patients had brain metastases already at the time of diagnosis, representing 14.3% of all and 33% of metastatic patients. EGFR mutations were present in 26 (29%) patients with BM.

We identified 168 patients who had BM at any time during their course of disease. Of these, 90 patients had metastases in CNS already at the time of diagnosis and 78 patients progressed to CNS during the treatment and the course of the disease. Out of 168 patients with BM, 47 had EGFR activating mutations (28%). The median follow-up time was 53 months. The data on the basic characteristics of this subgroup of patients (separately for those with BM at diagnosis and for those who developed BM later) are presented in Table 2. The median age at diagnosis for patients with BM at diagnosis was 61.5 years and did not differ due to EGFR status. The proportion of women was higher among EGFR positive patients (69.2%), yet this was not statistically significant compared to EGFR negative patients (MW-U test, p = 0.127). At diagnosis, only 3 patients (11.5%) with EGFR mutated tumours had BM as the only metastatic site, while there were 22 (34%) such patients in EGFR wild type tumours (p = 0.029). No such difference was seen in patients who had BM later during the course of the disease (p = 0.440). EGFR wild type patients in stage I–III progressed to CNS more often than EGFR mutant patients, p < 0.001.

### Cumulative incidence of BM

**FIGURE 1.**
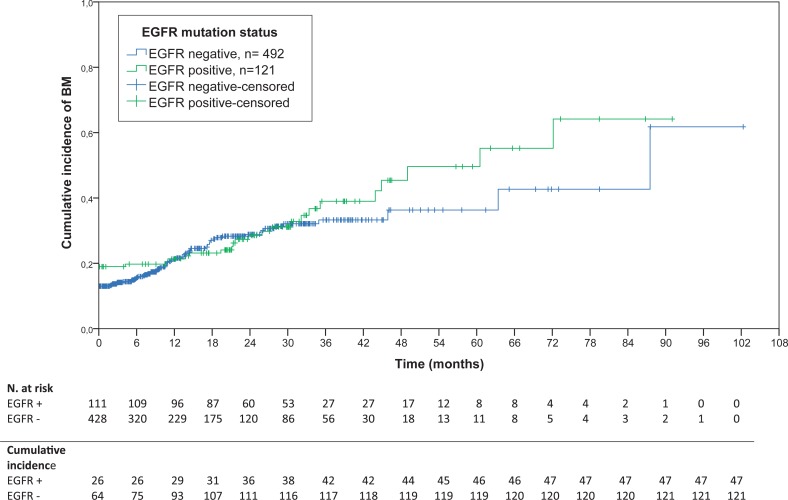
Cumulative incidence of BM in all adenocarcinoma patients by EGFR status.

The cumulative incidence of BM for all 629 patients analysed is presented in Figure 1. The incidence of BM did not differ among EGFR groups, the log rank p = 0.47. While more EGFR positive than negative patients had BM already at diagnosis (19% *vs.* 13%), the difference was only marginally significant (MW-U, p = 0.078).

Metastases developed after a median time of 14.3 months (CI 13.2 – 15.4) in 78 patients who had no BM at diagnosis. This group was not homogenous with regard to stage, there were 45 nonmetastatic and 33 metastatic patients, but this did not influence the TDBM. The median time to CNS progression for EGFR mutated patients was much longer than for EGFR wild type patients, 25.8 *vs.* 11.8 months (log rank, p = 0.002).

### Specific oncological treatment before the development of BM

All 78 patients without BM at diagnosis had a specific oncological treatment of primary tumour. The patients with non-metastatic disease at diagnosis (45 patients) received various combinations of surgery, radiotherapy and chemotherapy. According to guidelines, none received TKI as a primary treatment. Ten patients (3 EGFR positive) had only a surgical treatment of the primary tumour and CNS was the first site of disease progression in 5 patients, none of them EGFR positive. Six patients were treated with radiotherapy only, among them only one was EGFR mutant and received an intermittent treatment with chemotherapy and TKI at the first progression, which was not to CNS, and developed BM while on maintenance treatment with TKI. Thirty patients had a multimodality treatment, two were EGFR mutant. Metastatic patients received systemic treatment, either chemotherapy or TKI. In fact, 12 (80%) of all 15 EGFR positive patients received TKI already as a first line treatment.

In summary, all 21 EGFR positive patients without BM at diagnosis actually received treatment with TKI either as an initial or one of subsequent therapies, all before the development of BM.

### Specific oncological treatment after the diagnosis of BM

Out of 90 patients with BM at diagnosis, 66 (73%) patients started their treatment with whole brain radiotherapy (WBRT). After WBRT, all 17 EGFR positive patients received TKI treatment. Among 49 EGFR negative patients, 4 were also given TKI, chemotherapy was administered to 25 patients and best supportive care (BSC) to 20 patients. In the group of 24 patients who never had cranial irradiation, there were 9 EGFR positive patients, 7 received TKI and 2 BSC only. EGFR wild type patients in the group without WBRT received chemotherapy (7), TKI (1) and BSC (7). Altogether, no systemic therapy was delivered to 29 (32%) patients (2 EGFR positive) who had BM already at the diagnosis.

Within the group of 78 patients who developed BM later, 63 (80%) had WBRT and afterwards 40 patients (63%) received no systemic treatment, including 7 EGFR positive patients. Of the remaining 10 EGFR positive patients, 9 received TKI and 1 had chemotherapy. Among 15 patients without irradiation, BSC was given to 13 patients and TKI to 2 EGFR positive patients.

In summary, WBRT was delivered to 128 (76%) out of 168 patients with BM, while 40 patients had no irradiation of CNS. In comparison to EGFR wild type patients, those with EGFR mutations treated with WBRT had a longer TTD, 6.9 *vs.* 2.6 months (log rank, p = 0.005). Among all, 11 patients had a brain metastases resection followed by irradiation. We grouped patients into 3 categories regarding the dose of WBRT delivered (< 20 Gy, 21–30 Gy or > 30 Gy). The patients receiving a higher dose had a statistically significant longer TTD (log rank, p = 0.005). This difference was even more pronounced within each dose group for EGFR positive patients; however, no statistic was computed due to the small number of cases in some groups. Patients without WBRT had a statistically lower TTD (log rank, p = 0.002).

The systemic treatment resulted in a longer survival after BM compared to no systemic treatment, though only one half of the patients (52%) received one. The curves for TKI and chemotherapy overlap and show no meaningful difference. Treatment with TKI after the diagnosis of BM was administered to 49 patients (71% were EGFR positive) and chemotherapy to 37 (97% EGFR negative) patients.

### Median survival time from diagnosis of BM (TTD)

**FIGURE 2.**
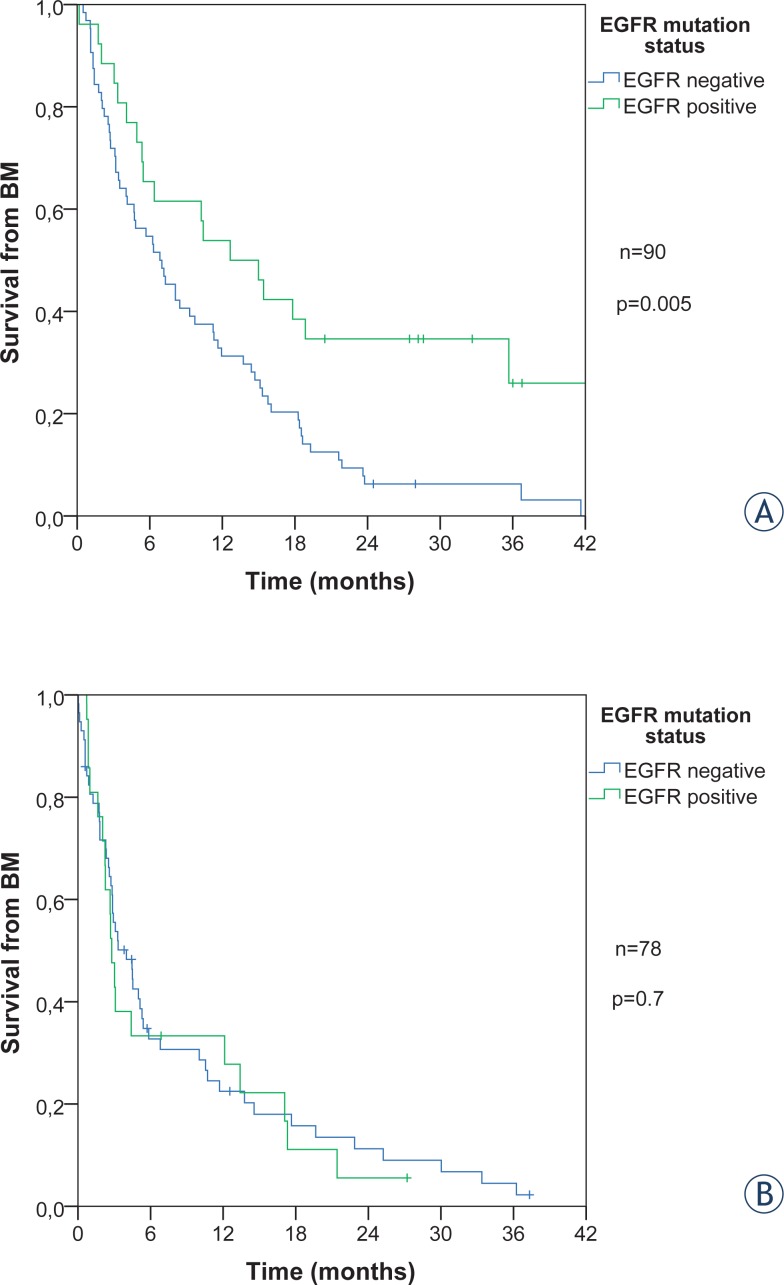
Survival from diagnosis of BM according to EGFR status: **(A)** for patients with BM at diagnosis and **(B)** for patients who developed BM later during the course of the disease.

The TTD for all 168 patients with BM was 5.3 months (CI 3.9–6.6). EGFR positive patients had a longer TTD as compared to EGFR negative patients, 6.3 *vs.* 4.8 months, respectively (log rank, p = 0.026). This difference is entirely due to a better survival of EGFR positive patients who had BM at initial diagnosis (12.6 months for EGFR positive and 6.8 for EGFR negative patients, p = 0.005). In those patients who developed BM later, the TTD was significantly shorter (3.1 months) and there was no significant difference between EGFR positive and negative patients (p = 0.7) (Figure 2).

Table 3 presents the results of univariate and multivariate analysis of survival from the date of BM (TTD) for patients with BM at diagnosis and those who developed BM later. The TTD for patients with BM at diagnosis (90 patients) was influenced by the EGFR status, age, weight loss, PS, WBRT and systemic treatment according to univariate analysis. The multivariate analysis showed that beside EGFR status also PS, WBRT and systemic treatment were significant.

In the TTD for patients who developed BM later (78 patients) age, systemic treatment and WBRT were significant in univariate, but only systemic treatment in multivariate Cox analysis. The EGFR status showed no significance in patients who developed BM during their course of disease.

### Overall survival time

**FIGURE 3.**
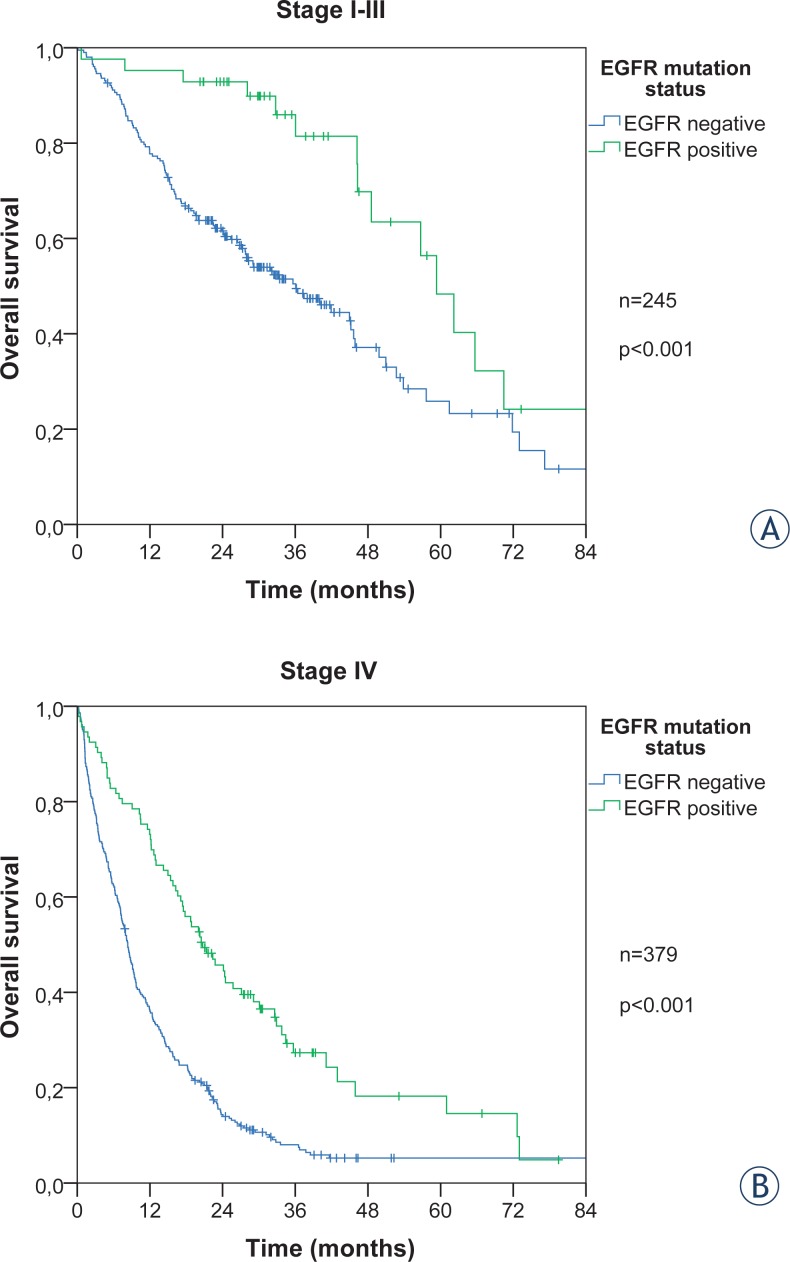
Overall survival for EGFR positive and negative patients in stages I–III **(A)** and IV **(B)**.

The overall survival of patients with EGFR activating mutations among all 629 adenocarcinomas was significantly longer regardless of metastatic status, log rank p < 0.001 (Figure 3). The median survival time for stage I–III was 59 months for EGFR positive and 36 months for EGFR negative patients. Metastatic patients had a shorter median survival, 20.6 months for EGFR mutant and 8.3 months for EGFR wild type.

**FIGURE 4.**
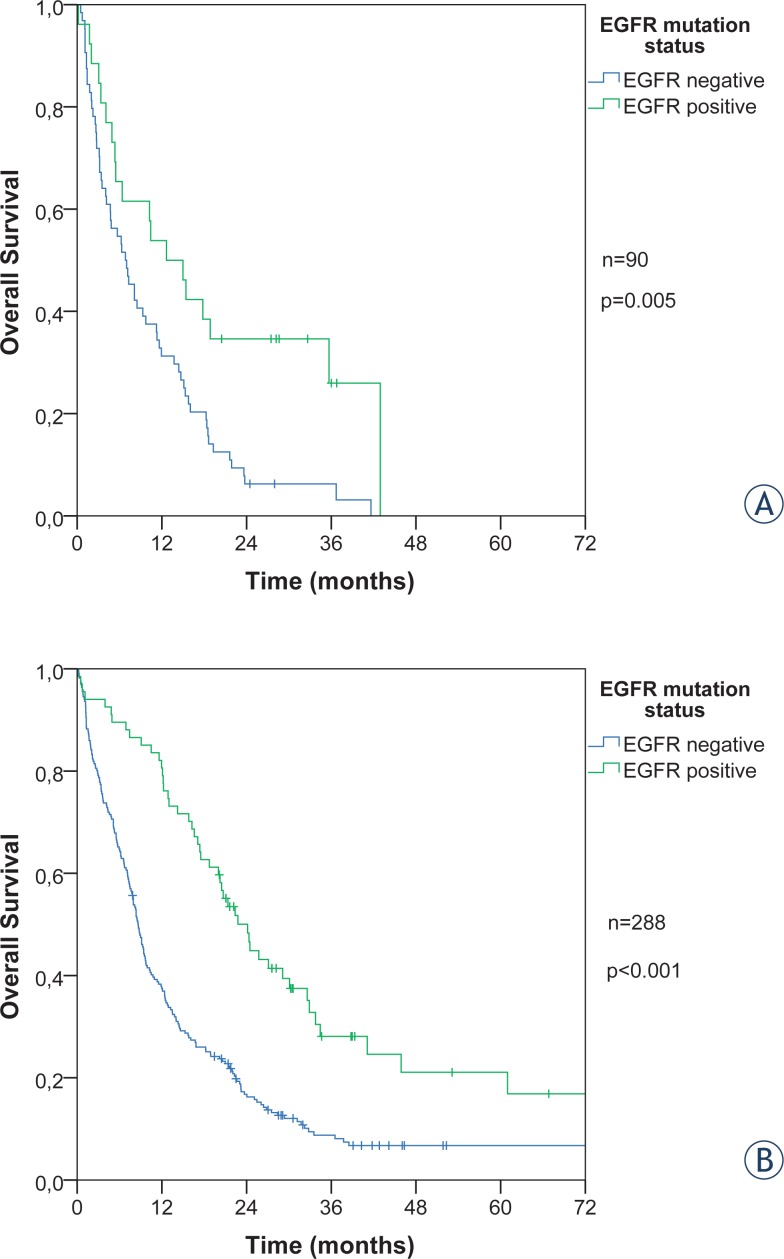
Overall survival by EGFR status for metastatic patients with brain metastases at diagnosis **(A)** and those without them **(B)**.

The presence of BM at the diagnosis of metastatic disease was a clear negative prognostic factor. The patients who had a metastatic disease at diagnosis, yet not to CNS, had a longer median survival compared to the patients with a metastatic disease to CNS at diagnosis (10.7 *vs.* 7.3 months). The difference within those two subgroups also persists in accordance with the EGFR status. The survival was twice longer in EGFR mutated (24.1 *vs.* 12.6 months) than in wild type patients (8.6 *vs.* 6.8 months) (Figure 4).

## Discussion

Our retrospective analysis belongs to the largest reports on nationally-based lung adenocarcinoma tested for EGFR mutations. We found that 28% of adenocarcinoma patients developed BM at any time during their course of disease. The majority of papers report a frequency of BM from 25 to over 50% for NSCLC, emphasizing a higher incidence in non-squamous histology.^1^,^2^,^8^–^13^ There are also some reports focusing exclusively on adenocarcinoma, yet the number of patients in these studies is low.^14^–^16^

The publications in recent years also include information on the EGFR status. Due to the increased prevalence of EGFR mutations in Asian population (30–40%) as compared to Caucasians (10–20%), the papers including a substantial proportion of Asian patients should be interpreted with caution since it has been reported that the incidence of BM is influenced by the EGFR status.^15^–^23^ Saad *et al*. reported no increased risk for development of BM with EGFR expression.^24^ All patients in our analyses were Caucasians.

The proportion of women in our analysis is high (52%) and does not reflect the epidemiological data (31%).^25^ The reason is a selection bias. The treating oncologists more often ordered EGFR testing at relapse for women and non-smokers, since all publications report a higher probability of EGFR mutations in those subgroups of patients.

In our analysis, the BM were present in 90 (14%) patients already at diagnosis. The same proportion was reported by Sekine *et al.* for 174 analysed Asian patients of whom 40% were EGFR mutated.^20^ The proportion of our patients with BM at diagnosis among all EGFR positive ones compared to all EGFR negative ones was higher, yet it did not reach statistical significance (19% *vs.* 13%, log rank, p = 0.078). Fujiwara reported this proportion to be 32% in the EGFR positive and 27% in the EGFR negative group among 141 analysed Asian patients.^15^

In our series, the BM was the only metastatic site for 25 patients at diagnosis, of whom only 3 were EGFR positive (p = 0.029). Significantly more isolated BM in EGFR negative patients was also found in the study of Eichler *et al.*, 31% *vs.* 7% (p = 0.03).^25^ On the contrary, Lee *et al*. found a higher incidence of isolated BM in EGFR mutated patients from a series of 117 resected tumours (24% *vs.* 9%), which, however, was not statistically significant.^21^ This finding indicates a different biology of the disease. It is therefore possible that patients with EGFR mutations are more prone to metastasing, including CNS, or they produce more often asymptomatic metastases and, consequently, patients are diagnosed at a later stage. One can also speculate that women nonsmokers (the majority of EGFR positive patients), although having medical and breathing problems, are not considered being at risk of having lung cancer in spite of seeking medical attention relatively early.

As shown in Figure 1, in the first year the curve of cumulative incidence of BM in EGFR mutated patients rises slower than in EGFR wild type, yet after one year the curves of EGFR mutant and EGFR wild type tumours overlap. Our patients without BM at diagnosis progressed to CNS after a median time of 14.3 months. The interval was longer for EGFR positive patients *vs.* EGFR negative ones, 25.9 *vs.* 11.9 months, respectively (p = 0.002). We believe that this observation is entirely due to a longer survival of EGFR mutated patients since a substantial proportion of EGFR wild type patients die already within the first year and never have a chance to develop BM. The time to the development of BM was also longer in EGFR positive patients in the study by Eichler *et al*., 19 *vs.* 14 months, yet this was not statistically significant.^25^

The median survival time from the diagnosis of brain metastases to death (TTD) was 5.3 months for all patients with BM. The EGFR mutation status strongly influenced the median survival time if BM had been discovered already at diagnosis (12.6 *vs.* 6.8 months) with no significant impact on those found later during the course of disease. A difference in accordance with the EGFR status was also found by Eichler *et al*., 14.5 *vs.* 7.6 months (p = 0.09).^25^ The TTD for our EGFR positive patients compares favourably to 5.5 months reported by Heon.^26^ Another report including 70% of EGFR positive patients found an overall survival from BM onset to be 15 months.^27^ A favourable survival of EGFR mutated patients with BM (13.2 *vs.* 6.8 months, p = 0.001) was also reported by Hsiao.^28^

Patients treated with WBRT had a longer TTD than those without it, which was also reported in other studies.^17^,^29^ EGFR positive patients had a longer TTD within the irradiated and the non-irradiated group as compared to EGFR negative patients. Gow *et al.* also reported that patients with EGFR mutations and WBRT had a better survival and response rate in univariate but only a trend in multivariate analysis.^30^ Additionally, we found that a higher dose led to longer survival. A combination of BM resection and postoperative WBRT did not result in a better TTD than WBRT alone for EGFR positive *vs.* negative patients, although the numbers are small. A systemic treatment delivered after the diagnosis of BM also increased survival and there was no difference whether patients received TKI or chemotherapy. Our finding is in accordance with a recent publication by Komatsu *et al*., who report a significant improvement in PFS and OS for patients treated with TKI after WBRT.^29^

Surprisingly, WBRT was an independent factor for better survival only in patients who had BM already at the time of diagnosis, while it had no influence on the subgroup of patients who developed BM later during the treatment and the course of disease. On the contrary, systemic treatment with chemotherapy or TKI had a significant influence on the survival of patients with BM of both subgroups. It is possible that the disruption of blood brain barrier by WBRT in patients with BM at diagnosis increased the permeability and penetration of TKI to CNS, leading to a prolonged survival; the mechanism was proposed by Ceresoli.^31^ All 78 patients who developed BM during the treatment and the course of disease in our study had one or more previous treatment lines with TKI before the BM onset. Due to the retrospective nature of our analysis, we could not establish any reliable PS at the time of BM from our medical records, therefore this was not included in the analysis. Usually, patients after several progressions and chemotherapy or TKI lines have a poor performance, which is also reflected in the fact that 63% of the patients only received BSC after WBRT. The reason for the non-effective WBRT might also be a lower total dose with shorter fractionation delivered to the majority of those patients.

EGFR mutated cell lines exposed to ionizing radiation *in vitro* show a 500 to 1000-fold reduced clonogenic survival.^32^ On the other hand, there are also *in vitro* reports for increased radioresistance of EGFR cell lines.^33^ It is believed that cells with EGFR mutations are radiosensitive and cells with EGFR overexpression are radioresistant.

Tanaka showed a strong *in vitro* effect of enhanced radiation response with gefitinib due to a prolonged double strand break and suppressed cellular DNA repair capability.^34^ TKI is considered to be a radiosensitizer, therefore TKI delivered concomitantly with WBRT represents one option of improved response rate (RR) in treating BM. The combination of WBRT and concomitant treatment with TKI remains controversial. While some researchers found no evidence of increased toxicity, others report an excellent RR and an increased OS at the expense of significant toxicity.^35^–^39^ Currently, TKI delivered concomitantly with WBRT is only recommended in clinical trials.

TKI alone was also used to treat asymptomatic BM from lung adenocarcinoma with high response rate of almost 70% in unselected Asian population of nonsmokers.^40^–^46^ In spite of all publications so far, the association between WBRT, the treatment with TKI and EGFR status is still unclear.

On the basis of the above findings, it is not unexpected that some investigators have proposed prophylactic cranial irradiation (PCI) for EGFR positive NSCLC patients.^47^ None of the PCI studies in NSCLC has so far demonstrated an improved OS, therefore this is not a routine practice as in small-cell lung cancer, although studies have been able to show a reduced incidence and delayed appearance of BM by 50%. There have been no reports of EGFR status impact on those parameters.^48^–^51^ Therefore we are eagerly awaiting the results of a prospective clinical trial going on in Germany; an outline was presented at ASCO 2012.^52^ Ongoing clinical trials are already focusing on new molecular targets, therefore retrospective real life analyses, although without possibility to omit all disadvantages of retrospective studies, could add to understanding this complex and disabling medical condition.

## Conclusions

Our results show that EGFR positive patients have a higher frequency of BM already at diagnosis, although not a statistically significant one, and a longer median survival than EGFR wild type patients. They develop BM later than EGFR negative patients, regardless of the stage and the previous treatment. The median survival of patients who develop BM during their course of disease is not different with regard to their EGFR status. Systemic treatment (either chemotherapy or TKI) was the only independent factor increasing the survival after the development of BM.

## Figures and Tables

**TABLE 1.** Patients characteristics

**Characteristics**	**Total patient number all**	**(%)**	**EGFR wild type**	**(%)**	**EGFR mutant**	**(%)**	**p**
**EGFR status**	**n = 629**	**100**	**492**	**78.2**	**137**	**21.8**	
**Age (years)**							
median	64		63		66		
range	25–88		25–87		36–88		
**Gender**							< 0.001
male	326	51.8	282	57.3	44	32.1	
female	303	48.2	210	42.7	93	67.9	
**Smoking status**							< 0.001
current	272	43.2	255	51.8	17	12.4	
former	181	28.8	152	30.9	29	21.2	
never	147	23.4	64	13.0	83	60.6	
no data	29	4.6	21	4.3	8	5.8	
**Performance status (WHO)**							0.513
PS0	84	13.4	63	12.8	21	15.3	
PS1	379	60.3	301	61.2	78	56.9	
PS2	97	15.4	72	14.6	25	18.2	
PS3	41	6.5	34	6.9	7	5.1	
PS4	4	0.6	2	0.4	2	1.5	
no data	24	3.8	20	4.1	4	2.9	
**Weight loss**							0.511
yes	183	29.2	141	28.7	42	30.6	
no	380	60.4	302	61.3	78	56.9	
unknown	66	10.4	49	10.0	17	12.5	
**Stage**							0.070
I–III	245	38.9	203	41.2	42	30.6	
IV	379	60.2	286	58.1	93	67.8	
undetermined	5	< 1	4	< 1	1	< 1	

EGFR = epidermal growth factor receptor

**TABLE 2.** Baseline characteristics of patients with brain metastases

	**Patients with BM at diagnosis**							**Patients who developed BM later**						

**Characteristics**	**Total patient number all**	**(%)**	**EGFR wild type**	**(%)**	**EGFR mutant**	**(%)**	**p**	**Total patient number (BM)**	**(%)**	**EGFR wild type**	**(%)**	**EGFR mutant**	**(%)**	**p**
**EGFR status**	**n = 90**	**100**	**64**	**71.1**	**26**	**28.9**		**n = 78**	**100**	**57**	**73**	**21**	**27**	
**Age (years)**														
median	61.5		60		66		0.346	59		59		59		0.154
range	38–87		38–81		40–87			(36–81)		(43–81)		(36–74)		
**Gender**														
male	39	43.3	31	48.4	8	30.8	0.127	41	52.6	31	54.4	10	47.6	0.598
female	51	56.7	33	51.6	18	69.2		37	47.4	26	45.6	11	52.4	
**Stage**														
I–III	NA		NA		NA		NA	45	57.6	40	70.1	5	23.8	0.000
IV	90	100	64	71.1	26	28.9		32	42.4	17	29.9	16	76.2	
**Metastatic sites**														
brain only	25	27.8	22	34.4	3	11.5	0.029	50	64.1	38	66.7	12	57.1	0.440
multiple sites	65	72.2	42	65.6	23	88.5		28	35.9	19	33.3	9	42.9	

BM = brain metastases; EGFR = epidermal growth factor receptor

**TABLE 3.** Univariate and multivariate analysis

	**TTD 90**			**TTD 78**		
	**7.3 months (CI 4.1–10.5)**			**3.1 months (CI 1.7–4.4)**		
	**Univariate**	**Multivariate**		**Univariate**	**Multivariate**	
	**p-value**	**p-value**	**HR (95% CI)**	**p-value**	**p-value**	**HR (95% CI)**
**Gender**						
(female/male)	0.24	-		0.81	-	
**Age**						
(< 61 / > 61)	0.15	0.09	ns	0.05	0.15	NS
**Smoking**						
(never/ever)	0.33	-		0.64	-	
**Weight loss**						
(no/yes)	0.04	0.09	NS	N/A	-	
**PS**			1.96			
(0–1/2–4)	0.00	0.01	(1.16–3.30)	N/A	-	
**EGFR**	0.00	0.00	0.37	0.70	-	
(negative/positive)			(0.18–0.77)			
**Systemic treatment**	0.00	0.00	4.32	0.00	0.00	2.16 (1.22–3.82)
(yes/no)			(2.39–7.82)			
**WBRT**	0.07	0.04	0.53	0.92	-	NS
(yes/no)			(0.28–0.99)			

EGFR = epidermal growth factor receptor; HR = hazard ratio; NS = not significant; PS = performance status; WBRT = whole brain radiotherapy
